# *Saccharomyces cerevisiae* Differential Functionalization of Presumed *ScALT1* and *ScALT2* Alanine Transaminases Has Been Driven by Diversification of Pyridoxal Phosphate Interactions

**DOI:** 10.3389/fmicb.2018.00944

**Published:** 2018-05-14

**Authors:** Erendira Rojas-Ortega, Beatriz Aguirre-López, Horacio Reyes-Vivas, Martín González-Andrade, Jose C. Campero-Basaldúa, Juan P. Pardo, Alicia González

**Affiliations:** ^1^Departamento de Bioquímica y Biología Estructural, Instituto de Fisiología Celular, Universidad Nacional Autónoma de México, Mexico City, Mexico; ^2^Laboratorio de Bioquímica-Genética, Instituto Nacional de Pediatría, Secretaría de Salud, Mexico City, Mexico; ^3^Departamento de Bioquímica, Facultad de Medicina, Universidad Nacional Autónoma de México, Mexico City, Mexico

**Keywords:** paralogous genes, pyridoxal phosphate binding, alanine transaminases, functional diversification, structural organization, alanine metabolism, phylogenetic analysis

## Abstract

*Saccharomyces cerevisiae* arose from an interspecies hybridization (allopolyploidiza-tion), followed by Whole Genome Duplication. Diversification analysis of *Sc*Alt1/*Sc*Alt2 indicated that while *Sc*Alt1 is an alanine transaminase, *Sc*Alt2 lost this activity, constituting an example in which one of the members of the gene pair lacks the apparent ancestral physiological role. This paper analyzes structural organization and pyridoxal phosphate (PLP) binding properties of *Sc*Alt1 and *Sc*Alt2 indicating functional diversification could have determined loss of *Sc*Alt2 alanine transaminase activity and thus its role in alanine metabolism. It was found that *Sc*Alt1 and *Sc*Alt2 are dimeric enzymes harboring 67% identity and intact conservation of the catalytic residues, with very similar structures. However, tertiary structure analysis indicated that *Sc*Alt2 has a more open conformation than that of *Sc*Alt1 so that under physiological conditions, while PLP interaction with *Sc*Alt1 allows the formation of two tautomeric PLP isomers (enolimine and ketoenamine) *Sc*Alt2 preferentially forms the ketoenamine PLP tautomer, indicating a modified polarity of the active sites which affect the interaction of PLP with these proteins, that could result in lack of alanine transaminase activity in *Sc*Alt2. The fact that *Sc*Alt2 forms a catalytically active Schiff base with PLP and its position in an independent clade in “sensu strictu” yeasts suggests this protein has a yet undiscovered physiological function.

## Introduction

*Saccharomyces cerevisiae* (*S. cerevisiae*) genome sequence revealed the presence of 26% duplicated genes suggesting that the *S. cerevisiae* lineage arose from a whole genome duplication (WGD), making this organism a suitable model to study diversification of duplicated genes (Gu et al., 2003; Kellis et al., 2004). A recent phylogenenomic study found compulsory evidence which allowed the proposition of an alternative origin for the *S. cerevisiae* lineage via an interspecies hybridization between two strains differentially related to the *Kluyveromyces, Lachancea* and *Eremothecium* (KLE) clade and the one related to *Zygosaccharomyces rouxii* and *Torulaspora delbrueckii* (ZT). Although it has not been demonstrated if the hybrid resulted from the fusion of two diploid cells, or two haploid cells that underwent a WGD, both possibilities could result in the formation of an organism with two copies of each gene of the *S. cerevisiae* genome. After the allotetraploid was formed, various recombination events, gene conversion, selective gene loss and selection pressures assembled *S. cerevisiae* genome acquiring the organization we observe today, which harbors conserved blocks of duplicated genes (Marcet-Houben and Gabaldón, 2015). Retained paralogous genes, can either provide increased dosage of the same product or subfunctionalize through a process, in which both copies of the gene lose a subset of their ancestral functions, acquiring new properties (Avendaño et al., 1997; DeLuna et al., 2001; Quezada et al., 2008; Colón et al., 2011; López et al., 2015; González et al., 2017). Neofunctionalization, results in the development of a totally new function (Merhej et al., 2015). Several models have been put forward to explain the functional evolution of gene copies. The duplication-degeneration-complementation model has been considered to explain cases in which functional diversification has resulted in the retention of both copies to carry out the function present in the original gene (Avendaño et al., 1997; Force et al., 1999; DeLuna et al., 2001; Quezada et al., 2008; Colón et al., 2011; López et al., 2015; González et al., 2017). The model known as “escape from adaptive conflict” posed by Hughes (1994) proposes that when the original gene performs two functions that cannot not be independently improved, after duplication each copy could be independently driven by positive selection to further develop one of the two functions. Transaminases represent an ideal model to study diversification of ohnologous genes carrying out two functions which are both needed to warrant metabolite synthesis, and which cannot be differentially meliorated (Colón et al., 2011; González et al., 2017). For the branched chain transaminases that determine biosynthesis and catabolism of isoleucine, leucine and valine (*Sc*Bat1/*Sc*Bat2), functional diversification was acquired through the acquisition of peculiar expression patterns and subcellular localization, resulting in the distribution of the two metabolic roles which are present in the ancestral type yeast *Kluyveromyces lactis* (*K. lactis*) (Colón et al., 2011) resulting in *Sc*Bat1 specialization for biosynthesis and *Sc*Bat2 for catabolism (Colón et al., 2011; González et al., 2017).

Analysis of the role of *Sc*Alt1 and *Sc*Alt2 in alanine metabolism has shown that *Sc*Alt1 is the first alanine aminotransferase found in yeast, which is capable of synthesizing and catabolizing alanine (García-Campusano et al., 2009), constituting the only known pathway for alanine catabolism, while *Sc*Alt2 has no alanine transaminase activity and thus has completely lost its role in alanine metabolism (Peñalosa-Ruiz et al., 2012). Since *Scalt1*Δ mutants do not require alanine, other alanine biosynthetic pathways must operate furnishing this amino acid. Accordingly, it has been proposed that in *S. cerevisiae*, the action of the glutamine-pyruvate transaminase and the ω-amidase pathway could constitute an alternative alanine biosynthetic route (Soberón et al., 1989), that would not play a catabolic role, since the reaction catalyzed by glutamine transaminase is irreversible (Soberón et al., 1989). In regard to ancestral-type yeasts, previous results from our laboratory (Escalera-Fanjul et al., 2017) have shown that *Lacchancea kluyveri* (*L. kluyveri*) and *Kluyveromyces lactis* (*K. lactis*) alanine transaminases; *LkAlt1* and *KlAlt1* are *ScAlt1* orthologous counterpart. Additionally, these two yeasts respectively display *Lk*Alt1 and *Kl*Alt1 alanine transaminase activity and unidentified alanine biosynthetic and catabolic pathways. In addition, the phenotypic analysis of *Lkalt1*Δ and *Klalt1*Δ null mutants showed that these proteins have a metabolic role, not related to alanine metabolism, since *Lkalt1*Δ does not achieve wild type growth rate. Thus, the ancestral alanine transaminase function (Escalera-Fanjul et al., 2017) was kept by the *ScAlt1*, which specialized its catabolic character, and lost the alanine independent role observed in the ancestral type enzymes. The fact that *Sc*Alt2 and *Sc*Alt1 conserve high identity with *Lk*Alt1 and *Kl*Alt1, indicates that *Sc*Alt1 and *Sc*Alt2 diversified after the ancestral hybrid was formed. It could be considered, that *Sc*Alt2 diversification resulted in loss of both alanine transaminase activity and the additional alanine-independent *Lk*Alt1 function. It can thus be concluded that *Lk*Alt1 and *Kl*Alt1 functional role as alanine transaminases was delegated to *Sc*Alt1, while *Sc*Alt2 lost this role during diversification, specializing a yet uncovered activity, which does not represent the additional alanine-independent activity observed in *Kl*Alt1 and *Lk*Alt1 since *ScALT2* is unable to complement both: *Lkalt1*Δ and *Klalt1*Δ mutants. Results suggest that *ScALT1* and *ScALT2* diversification pattern could fit the specialization or escape from adaptive conflict posed by Hughes (1994), although since *Sc*Alt2 function has not been identified, subfunctionalization or neo-functionalization cannot be ruled out. Identification of *Sc*Alt2 function and determination of whether it is present in ZT and or KLE will be necessary in order to address this matter.

The study presented in this paper has been aimed to carry out the structural characterization of *Sc*Alt1 and *Sc*Alt2 in order to better understand how *Sc*Alt2 functional diversification, led to loss of alanine transaminase activity. Our results show that *Sc*Alt2 has a more expanded structure in comparison to that displayed by *Sc*Alt1. This differential conformation could influence the polarity of the environment surrounding the active site, affecting the interaction of the catalytic site with PLP. Under physiological conditions, PLP interaction with *Sc*Alt1 allows the formation of two tautomeric PLP forms (enolimine and ketoenamine), while *Sc*Alt2 preferentially forms the ketoenamine PLP tautomer, indicating *Sc*Alt2 has different interaction patterns with PLP as compared to those displayed by *Sc*Alt1. However *Sc*Alt2 conserved a PLP binding domain, which allows the formation of a catalytically active Schiff base, suggesting this protein could have a yet undiscovered PLP-dependent activity.

## Materials and Methods

### Phylogenetic Analysis

A total of 33 taxa were used in the analysis, including six ascomycetes as outgroups. Amino acid sequences of alanine transaminases of hemiascomycetes were obtained from the Fungal Orthogroups Repository^1^ and Phylome Data Base^2^ (Huerta-Cepas et al., 2014) databases, using *Sc*Alt1 sequence as query. Alignment was performed and phylogenetic tree was constructed with the Muscle method using the *MEGA* version 5.0 of the UPGMB algorithm^3^ (Hall, 2013), using the Maximum Likelihood (ML) method. Prior to ML analysis, best-fit models of amino acid substitution were selected with the Bayesian Information Criterion (BIC) as implemented by *MEGA* version 5.0. Based on the BIC, the model that best-fitted Alts evolution was the LG+G+I model. Robustness of ML tree topologies were tested by bootstrap analyses, with 1000 replicates each.

### *ScALT1* and *ScALT2* Cloning and Overexpression in *E. coli*

*Saccharomyces cerevisiae ScALT1* and *ScALT2* genes were PCR amplified respectively using the 1/2 deoxyoligonucleotide pair: FrwAlt1 (GCG CGC CAT ATG CAA TCT TCG CTA AAC GAC CTG C) and RvsAlt1 (CGC GCG CTC GAG CCC TTT TAT TCA GTC ACG GTA TTG G), and the 3/4 oligonucleotides FrwAlt2 (GCG CGC GCT AGC ATG ACA ATG ACA CAC CAA CAG G) and RvsAlt2 (CGC GCG CTC GAG TCA ATT ACG ATA CTT GCT GAA GAAGAA ATC) using genomic DNA of the CLA1 WT (*MATa ScALT1 ScALT2 ura3*Δ*leu2*Δ) strain as a template. For *ScALT1* amplification, the mitochondrial localization sequence was not included. PCR products and pET-28a (+) plasmids were NdeI/XhoI digested and ligated after gel purification. Ligations were transformed into the DH5α *Escherichia coli* (*E. coli*) strain. Plasmids were purified and, correct cloning was verified by sequencing. For *ScALT1* and *ScALT2* heterologous expression, the Rosetta 2^TM^ (DE3) *E. coli* strain (Novagen) was transformed. *ScALT1* selected clones were grown in LB medium with 50 μg ml^-1^ of kanamycin and 70 μg ml^-1^ of chloramphenicol, grown at 37°C with shaking (250 rpm). When cultures reached an OD of 0.6 at 600 nm, expression of the proteins was induced with 200 μmol L^-1^ of isopropylβ-D-1-thiogalactopyranoside (IPTG), incubated overnight at 30°C with shaking (250 rpm), harvested by centrifugation at 1100 *g* for 15 min, and the cellular pellet was stored at -70°C until used. To over-express *Sc*Alt2 in soluble form we carried out the modified San-Miguel protocol (San-Miguel et al., 2013), which allows the induction of the soluble form of proteins which in standard protocols are sequestered in inclusion bodies. Several clones were grown on LB medium with 50 μg ml^-1^ of kanamycin and 70 μg ml^-1^ of chloramphenicol and incubated at 30°C with shaking (250 rpm). When cultures reached an OD of 0.2 at 600 nm, protein was induced with 100 μmol L^-1^ of IPTG, incubated for 1 week at 4°C with shaking (250 rpm) and harvested by centrifugation at 1100 *g* for 15 min, the cellular pellet was stored at -70°C until used. Over-expression of either *Sc*Alt1 or *Sc*Alt2 was carried out in strains carrying HIS-tags on the amino terminal end of the protein.

### Obtention of Whole Cell Soluble Protein Extracts

The cellular pellet of *Sc*Alt1 was suspended in 10 ml of 30 mmol L^-1^ imidazol, 1 M NaCl, 50 mM K_2_HPO_4_, 1 mmol L^-1^ EDTA, 1 mmol L^-1^ dithiothreitol, 1 mmol L^-1^ phenylmethylsulfonylfuoride (PMSF), pH 8. Soluble extracts were obtained by sonication (Ultrasonic Processor Model: VCX 130) with a tip sonicator maintaining the tubes on ice; five cycles (70% amplitude, 1 s on and 1 s off for 1 min) with 1 min of incubation on ice between each cycle. After centrifugation at 1100 *g* for 20 min at 4°C, the supernatant was stored at 4°C. *Sc*Alt2 soluble extract was prepared by resuspending thawed cells in 10 ml of lysis buffer [2 M NaCl, 50 mM K_2_HPO_4_, plus one Complete Mini EDTA-free protease inhibitor cocktail tablet (Roche, Inc.) and 1 mM phenylmethylsulfonylfluoride (PMSF), pH 8]. Protein extracts were obtained by sonication as above described.

### Immobilized Metal Affinity Chromatography (IMAC)

To purify *Sc*Alt1 protein, the supernatant was loaded on an equilibrated nickel column (Ni-NTA Agarose, Quiagen), washed with 50 volumes of 30 mmol L^-1^ imidazol, 50 volumes of 50 mmol L^-1^ imidazol, and 10 volumes of 80 mmol L^-1^ imidazol. The protein was eluted with 300 mmol L^-1^ imidazol and stored at 4°C unit used. To purify *Sc*Alt2 protein, supernatant was also purified through an equilibrated nickel column (Ni-NTA Agarose, Quiagen), washed with 50 volumes of lysis buffer; afterward, the protocol described for *Sc*Alt1 purification was followed. *Sc*Alt1 and *Sc*Alt2 homogeneity of proteins was verified by denaturing with a polyacrylamide gel electrophoresis (12% SDS-PAGE) and the gel stained with Coomassie Blue. Proteins were 10-fold concentrated with Amicon^®^ Ultra-15 10K centrifugal filter devices, and then diluted to the original sample volume with assay buffer (50 mM K_2_HPO_4_, 4 mM Mg_2_Cl, 100 mM PLP, at pH 7.5) three “washing out” cycles were performed.

### Alanine Transaminase Enzymatic Assay

Enzymatic assay followed a previously reported protocol (Bergmeyer, 2012). The reaction mixture contained (50 mM K_2_HPO_4_, 20 mM alanine, 6 mM α-ketoglutarate, 250 μM NADH, 5 U ml^-1^ of lactate dehydrogenase, and 40 μM PLP pH 7.5). Control, assays were performed without alanine. Specific activity, was determined and the slope of the negative control was subtracted to that obtained with the complete assay. All assays were performed at 25°C in a Varian Cary 50 spectrophotometer, following the absorbance at 340 nm. Protein was determined according to the method described by Lowry et al. (1951), using bovine serum albumin as standard. Initial velocities were obtained at different concentrations of both substrates simultaneously for *Sc*Alt1, alanine was varied from 2.00 to 15.00 mM and α-ketoglutarate from 0.05 to 10.00 mM. The results were globally fitted to the rate equation of a ping-pong mechanism (Segel, 1993), using GraphPad Prism 6.00 (Software, Inc.).

$$V_{0}=(V_{\max}[A]\left[B\right])/(K_{mA}\left[B\right]+K_{mB}\left[A\right]+\left[A\right]\left[B\right]).$$

### Homology Modeling for *Sc*Alt1 and *Sc*Alt2

DNA sequence of *ScALT1* and *ScALT2* were retrieved from *S. cerevisiae* S288c chromosome XII (NCBI Reference Sequence: NC_001144.5) and S288c chromosome IV (NCBI Reference Sequence: NC_001136.10). The two genes, *ScALT1* and *ScALT2* encode proteins of 1024 and 1014 amino acids long, respectively. The Basic Local Alignment Search Tool (BLAST^4^) (Altschul et al., 1997) was used to find homologous protein structures in the Protein Data Bank. The crystal structure of the alanine aminotransferase structure from *Hordeum vulgare* (3TCM.pdb) was selected for the modeling procedure (Duff et al., 2012). The amino acid sequence comparison of *ScAlt1* and *ScAlt2* sequence comparison showed a 46 and 45% identity with a 94 and 96% coverage with the respective targets. *Sc*Alt1 and *Sc*Alt2 models were constructed using MODELLER 9.17 with 3TCM.pdb (Webb and Sali, 2017) file as template structure, models with lower energy were chosen. Finally, a simple structural refinement of full-atom was performed using Rosetta (Leaver-Fay et al., 2011) “relax” application. Models were validated using the Verify-3D (structure evaluation software) (Lüthy et al., 1992) and What check (protein verification tools software) (Hooft et al., 1996) computer programs. Ligand structure was constructed using HyperChem software (Froimowitz, 1993). The structures of the ligands were minimized using Gaussian 09, revision A.02 (Gaussian, Inc., Wallingford, CT, United States) at DTF B3LYP/3-21G level of theory.

### Circular Dichroism Analysis

*Sc*Alt1 and *Sc*Alt2 secondary structures were spectroscopically evaluated using circular dichroism (CD) with a spectropolarimeter (Jasco J-810^®^, Easton, MD, United States) equipped with a Peltier thermostated cell holder in a 1 mm path-length quartz cuvette. Far UV-CD spectra of *Sc*Alt1 and *Sc*Alt2 were obtained from 190 to 280 nm at 1 nm intervals using a protein concentration of 0.25 mg ml^-1^ in 50 mM K_2_HPO_4_, 4 mM Mg_2_Cl, 100 μM PLP at pH 7.5. Experiments were performed at 25°C.

### *Sc*Alt1 and *Sc*Alt2 Thermal Stability

Thermal stability of *Sc*Alt1 and *Sc*Alt2 was determined following the CD signal at 222 nm varying the temperature from 20 to 90°C at an increase at a rate of 1°C/2.5 min, and a protein concentration of 0.250 mg ml^-1^. Spectra without protein were subtracted from those that contained the recombinant *Sc*Alt1 and *Sc*Alt2 purified proteins, respectively. The fraction at which 50% of the protein was unfolded, corresponds to the melting temperature (*T_m_*) value.

### Intrinsic Fluorescence of *Sc*Alt1 and *Sc*Alt2 Proteins

Emission-fluorescence spectra of *Sc*Alt1 and *Sc*Alt2 proteins were recollected from 300 to 450 nm at an excitation wavelength of 295 nm with a Shimadzu RF5-00U spectrofluorometer. Enzyme concentration was 200 μg ml^-1^ in 50 mM K_2_HPO_4_, 4 mM Mg_2_Cl, 100 μM PLP at pH 7.5. Slope observed without protein was recorded and subtracted from samples containing purified *Sc*Alt1 or *Sc*Alt2. For the experiment performed with Gdn/HCl, enzyme was added to a 6 M solution of guanidinum chloride Gdn/HCl, prepared in 50 mM K_2_HPO_4_, 4 mM Mg_2_Cl, at pH 7.5. The emission spectra were recorded after 2 h of incubation with Gdn/HCl.

### Fluorescence Quenching

Emission-fluorescence spectra of *Sc*Alt1 and *Sc*Alt2 proteins were recorded from 300 to 450 nm at an excitation wavelength of 295 nm with a Shimadzu RF5-00U spectrofluorometer, using solutions having a protein concentration of 250 μg ml^-1^. The fluorescence of proteins monitored at their emission maxima, was quenched by the addition of small aliquots of an 8 M acrylamide solution, as described previously (Eftink and Ghiron, 1976), and analyzed with the Stern–Volmer equation:

$$F0/F=1+K_{sv}[Q].$$

In this equation, F0/F is the ratio of the fluorescence intensity in the absence and presence of a given concentration of quencher [Q], and *K*_sv_ is the quenching constant.

### Size Exclusion Chromatography

Molecular exclusion chromatography was performed with a Superose 6 10/300 GL column coupled to an Äkta FPLC system. The mobile phase had 50 mM K_2_HPO_4_, 4 mM Mg_2_Cl, pH 7.5. Protein elution was monitored by following the absorbance at 280 nm. Column was calibrated with Bio-Rad molecular weight standards.

### Determination of *Sc*Alt1 and *Sc*Alt2 Alanine Binding Affinity

The emission-fluorescence spectra of *Sc*Alt1 and *Sc*Alt2 were recorded from 300 to 450 nm at an excitation wavelength of 295 nm with a Shimadzu RF5-00U spectrofluorometer. The *Sc*Alt1 and *Sc*Alt2 alanine binding affinity was determined by fluorescence titrations. *Sc*Alt1 and *Sc*Alt2 (250 μg ml^-1^) were titrated by adding increasing alanine concentrations (0–50 mM) suspended in 50 mM K_2_HPO_4_, 4 mM Mg_2_Cl, 100 mM PLP pH 7.5, following the changes in fluorescence at 389 nm. Dissociation constant (Kd) values were computed by fitting the data to “One site- Specific binding equation”:

$$Y=\left(B_{\max}X\right)/\left(Kd+X\right).$$

### Determination of ScAlt1 and ScAlt2 Catalysis With Different Amino Acids

To analyze the catalysis of *Sc*Alt1 and *Sc*Alt2 with different amino acids we monitored the formation of an external Schiff Base, which is the first step of the catalysis of all PLP dependent enzymes. For this purpose we followed shifts in the UV-visible spectrum on NanoDrop^TM^ and compared the spectrum of the protein with and without amino acids. When PLP forms an external Schiff Base, the appearance of a maximum peak at 325 nm was observed (Binter et al., 2011). *Sc*Alt1 and *Sc*Alt2 were prepared at 12 μg/μl in 50 mM KH_2_PO_4_, 4 mM MgCl_2_, 500 mM PLP, 10 μl of the protein were mixed with 1 μl of a 10X stock solution of the corresponding amino acid. Amino acid stock solutions were prepared in KH_2_PO_4_ and 4 mM MgCl_2_. 10X amino acid stocks were prepared as follows: alanine 162 mM, arginine 60 mM, asparagine 8.2 mM, aspartic acid 81 mM, cysteine 238 mM, glutamic acid 81 mM, glutamine 171 mM, glycine 8 mM, histidine 20 mM, isoleucine 7 mM, leucine 5 mM, lysine 19 mM, methionine 1.6 mM, phenylalanine 5 mM, proline 2.3 mM, serine 23 mM, threonine 22 mM, tryptophan 2 mM, tyrosine 3 mM, and valine 361 mM.

### Absorption Spectroscopy

*Sc*Alt1 and *Sc*Alt2 UV-visible spectra were recorded on a Cary 400 UV-visible spectrophotometer (Varian) and analyzed using Cary WinUV software (Varian). Spectra were recorded in samples containing a 6 mg ml^-1^ of protein in 50 mM K_2_HPO_4_, 4 mM Mg_2_Cl, pH 7.5 and at room temperature. *Sc*Alt1 and *Sc*Alt2 spectra obtained in the presence of PLP were recorded immediately after the addition of 100 μM PLP.

### Reduction of the Schiff Base in the Presence of Sodium Borohydride

To reduce the Schiff base in *Sc*Alt1 and *Sc*Alt2, 6 mg ml^-1^ samples of purified *Sc*Alt1 and *Sc*Alt2 were suspended in 50 mM K_2_HPO_4_, 4 mM Mg_2_Cl, 100 μM PLP pH 7.5 and 15 μL of 1 M NaBH_4_ (dissolved in 50 mM K_2_HPO_4_, 4 mM MgCl_2_, pH 7.5 buffer) was added to reduce the Schiff base. The reduction reaction was allowed to proceed for 1 h in the dark. UV-visible spectra of *Sc*Alt1 and *Sc*Alt2 were registered (scan range: 300–600 nm) using a Cary 400 UV-visible spectrophotometer (Varian) before reduction to confirm the presence of PLP linked by a Schiff base and after reduction to confirm the presence of a reduced Schiff base (band at 325 nm). The protein was dialyzed against in 50 mM K_2_HPO_4_, 4 mM MgCl_2_, pH 7.5 buffer.

### PLP Quantification

Prior to the absorbance measurement, *Sc*Alt1 and *Sc*Alt2 were converted to the holo-form by adding PLP to a final concentration of 500 μM, and incubated for 1 h at 4°C. PLP excess was removed by purification using a Penefsky column (Penefsky, 1979). To perform UV-visible assays, 6 mg ml^-1^ of recombinant *Sc*Alt1 and *Sc*Alt2, were used after blanking the spectrophotometer with 50 mM K_2_HPO_4_, 4 mM MgCl_2_, pH 7.5 buffer. *Sc*Alt1 and *Sc*Alt2 (6 mg ml^-1^), were denatured with 6 M Gdn/HCl and incubated for 2 h at room temperature. UV-visible spectra of *Sc*Alt1 and *Sc*Alt2 were obtained (scan range: 300–600 nm) using a Cary 400 UV-visible spectrophotometer (Varian) before and after denaturation to confirm the presence of the Schiff base. To find out if PLP was irreversibly bound to the *Sc*Alt1 and *Sc*Alt2 proteins, the denatured protein was separated from the PLP using an Amicon Ultra-15 device. Then, the UV-visible spectra were obtained from the unbounded PLP that went through the filter; PLP quantification was obtained using a calibration curve. To find out whether the protein conserved bound PLP, the initial volume of the protein was reconstituted with 6 M Gdn/HCl and an absorbance scan at 300–600 nm was performed.

### Experimental Reproducibility

Representative results are presented, all the experiments were performed at least two or three times.

## Results

### ScAlt1 Displays a Kinetic Ping-Pong Mechanism Typical of Transaminases

**FIGURE 1:**
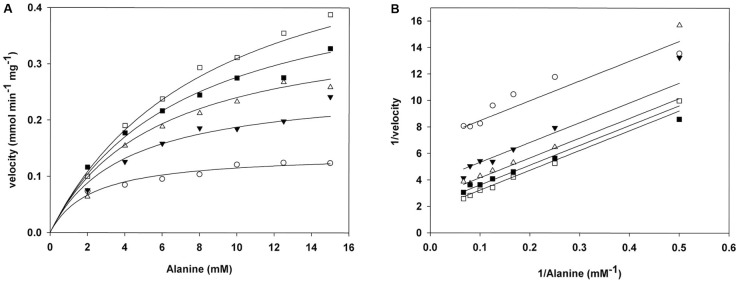
*Sc*Alt1 displays transaminase ping-pong kinetics. **(A)** Global fit of *Sc*Alt1 to ping- pong equation. Ka (alanine) 10.39 ( ± 1.12) mM, Kb (α-ketoglutarate) 0.67 ( ± 0.06) mM, Vmax 0.69 ( ± 0.04) mmol mg^-1^ min^-1^ R^2^ 0.98. **(B)** Double reciprocal plots of *Sc*Alt1 initial velocities as a function of increasing alanine at a fixed α-ketoglutarate concentration. White squares 0.2 mM α-ketoglutarate, black squares 0.4 mM α-ketoglutarate, white triangles 0.8 mM α-ketoglutarate, black triangles 1.5 mM α-ketoglutarate, white circles 4.0 mM α-ketoglutarate.

To investigate the kinetic properties of *Sc*Alt1 from *S. cerevisiae*, amino terminal His-tagged enzyme was purified to electrophoretic homogeneity by IMAC, after heterologous over-expression in *E. coli*, as described in Section “Materials and Methods.” *Sc*Alt2 protein was also purified (Supplementary Figure S1), and as previously reported, it did not show alanine transaminase activity (Peñalosa-Ruiz et al., 2012). For *Sc*Alt1, initial velocity measurements were performed varying α-ketoglutarate and alanine concentrations. The resulting double reciprocal plots (Lineweaver–Burk) for both substrates are consistent with a ping-pong mechanism (**Figure 1B**). The data were globally fitted to the ping-pong equation (**Figure 1A**). The resulting kinetic parameters are presented in **Table 1**. In addition, **Table 1** shows the kinetic parameters for two ancestral type yeasts: *Lk*Alt1 (*Lacchancea kluyveri*) and *Kl*Alt1 (*Kluyveromyces lactis*) (Escalera-Fanjul et al., 2017). *Sc*Alt1 showed a Km for alanine and α-ketoglutarate similar to those found for *Lk*Alt1 and *Kl*Alt1, while *Sc*Alt1*k*_cat_was two to threefold higher than the ones found in both of the ancestral-type species, this could be attributed to the fact that for *Sc*Alt1, purification was carried at 4°C, while those of *Lk*Alt1 and *Kl*Alt1 were performed at room temperature (25°C).

**Table 1:** Kinetic parameters of Alts of different yeasts.

	Ka alanine (mM)	Kb α-ketoglutarate (mM)	k_cat_ (s^-1^)	
*Sc*Alt1	10.39	0.67	698	This work
*Lk*Alt1	4.88	0.22	355	Escalera-Fanjul et al., 2017
*Kl*Alt1	17.22	0.92	205	Escalera-Fanjul et al., 2017


### Although *Sc*Alt1 and *Sc*Alt2 Share Catalytic Sites, *Sc*Alt2 Does Not Display Alanine Transaminase Activity

**FIGURE 2:**
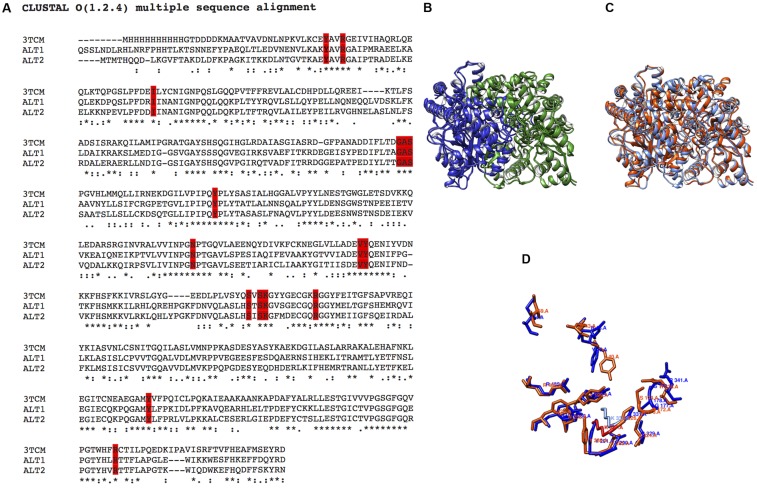
Sequence and structural alignment of *Sc*Alt1 and *Sc*Alt2. **(A)** Amino acid sequence alignment of *Sc*Alt1, *Sc*Alt2 and *Hordeum vulgare* alanine transaminase (3TCM). The residues involved in the alanine and PLP binding are highlighted in red. **(B)** Structural alignment of *Sc*Alt1 and *Sc*Alt2 protein models. Both models are constituted by dimeric proteins: subunit A in blue, subunit B in green. **(C)** Comparison of *Sc*Alt1 and *Sc*Alt2 protein models, *Sc*Alt1 in blue and *Sc*Alt2 in red. **(D)** Comparison of catalytic residues, *Sc*Alt1 in blue, and *Sc*Alt2 in red.

To determine whether *Sc*Alt2 contained the catalytic residues and the PLP presumed binding site, we performed amino acid sequence alignment with *Sc*Alt1 and *H. vulgare* (barley) alanine transaminases (**Figure 2A**) (Duff et al., 2012). Barley alanine transaminase is a dimeric PLP-dependent enzyme, which has been purified and kinetically characterized. The enzyme exhibits a ping-pong reaction mechanism, and its main physiological role is to catalyze the forward (alanine-forming) and the reverse (glutamate-forming) reactions. It can synthesize aspartate with 10% efficiency as compared to alanine and its crystallographic structure has been determined (Duff et al., 2012). Amino acid sequence comparison revealed that both, *Sc*Alt1 and *Sc*Alt2 conserve the amino acids which have been found to be involved in alanine and PLP binding (**Figure 2A**). The analysis showed that *Sc*Alt1 and *Sc*Alt2 conserved approximately 45% amino acid identity with *H. vulgare* whereas the identity between *Sc*Alt2 and *Sc*Alt1 was 67%. Furthermore, potential catalytic residues and the binding site region of the prosthetic group can be recognized in the three proteins (**Figure 2A**). This result suggests that *Sc*Alt2 should have transaminase activity. In addition, the alignment of the 3D models of *Sc*Alt1 and *Sc*Alt2 built on the *H. vulgare* (3TCM.pdb) template shows very similar topological orientation of their catalytic residues (**Figures 2B–D**). These results prompted us to carry out *Sc*Alt1 and *Sc*Alt2 structural analysis.

### *Sc*Alt1 and *Sc*Alt2, Display a Similar Structure; However, *Sc*Alt2 Has a More Expanded Organization, Probably Allowing Higher Solvent Accessibility

**FIGURE 3:**
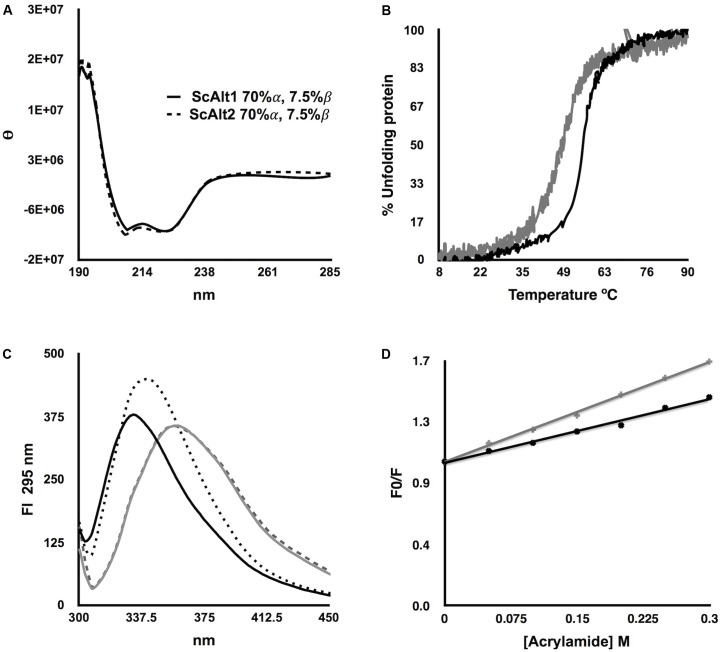
Structural Characterization of *Sc*Alt1 and *Sc*Alt2. **(A)** CD at far UV spectrum, shows *Sc*Alt1 and *Sc*Alt2 secondary structure. **(B)** Thermal denaturation of *Sc*Alt1 (black) and *Sc*Alt2 (gray). **(C)** Intrinsic Fluorescence in native and denaturing conditions: native *Sc*Alt1 continuous black line, denatured *Sc*Alt1 gray continuous line, native *Sc*Alt2 in black dotted line and denatured *Sc*Alt2 gray dashed line. **(D)** Acrylamide quenching (Stern–Volmer plot) of *Sc*Alt1 and *Sc*Alt2, the slope of the linear regression represents the Stern–Volmer constant or K_SV_. K_SV_ for *Sc*Alt1 black line = 1.48 m^-1^ R^2^ 0.99, and for *Sc*Alt2 gray line K_SV_ = 2.96 M^-1^ R^2^ 0.98.

Considering that the sequence alignment between *Sc*Alt1 and *Sc*Alt2 shows an identity of 67%, we studied the effect of such difference over their native structures. The secondary structure of the recombinant proteins was studied by monitoring the CD signal at far-UV (**Figure 3A**); the relative values of α-helix and β-sheet determined from the CD data are also depicted (**Figure 3A**, inset). No significant changes were detected between the CD signals or the secondary structure values of *Sc*Alt1 and *Sc*Alt2. In addition, the thermostability of both proteins in the presence of PLP was measured following the CD signal at 222 nm. *Sc*Alt1 showed a higher stability than that observed for *Sc*Alt2 (55°C *Sc*Alt1 vs. 49°C *Sc*Alt2) (**Figure 3B**).

We evaluated differences in the tertiary structure of proteins by following their intrinsic fluorescence under native and denaturing conditions (**Figure 3C**). Under native conditions both proteins are totally folded; the addition of the denaturant agent Gdn/HCl, promoted in *Sc*Alt1 and *Sc*Alt2 a 24 nm and 17 nm red-shift of the wavelength to obtain the maximal fluorescence peaks at 357 nm. Besides, under native conditions, different λ max for *Sc*Alt1 and *Sc*Alt2 were obtained (333 and 340 nm, respectively). These results might suggest differences in the fluorescence contribution of the aromatic residues between both proteins. Alternatively, the data may indicate differences in solvent accessibility in which *Sc*Alt1 showed less accessibility than *Sc*Alt2, explaining the 7 nm red-shift in the *Sc*Alt2 under native conditions.

To assess these assumptions, we performed a Stern–Volmer fluorescence quenching test of *Sc*Alt1 and *Sc*Alt2 by using acrylamide (**Figure 3D**). *Sc*Alt2 showed a higher Stern–Volmer quenching constant value (2.96 M^-1^) than *Sc*Alt1 (1.48 M^-1^), confirming that the former has higher solvent accessibility. Finally, to determine the quaternary structure, we performed size exclusion chromatography experiments. The results showed that both proteins elute as dimers. Considering that the theoretical molecular weight for the *Sc*Alt1 monomer is 61 kDa and for that of *Sc*Alt2 is 58 kDa the obtained experimental results, 147 kDa for *Sc*Alt1 and 127 kDa for *Sc*Alt2, indicate a dimeric organization (Supplementary Figure S2). Collectively, the data show that the global structure of *Sc*Alt1 and *Sc*Alt2 are very similar, although *Sc*Alt2 shows higher solvent accessibility.

### *Sc*Alt2 Does Not Bind Alanine, Confirming Its Lack of Alanine Transaminase Activity

**FIGURE 4:**
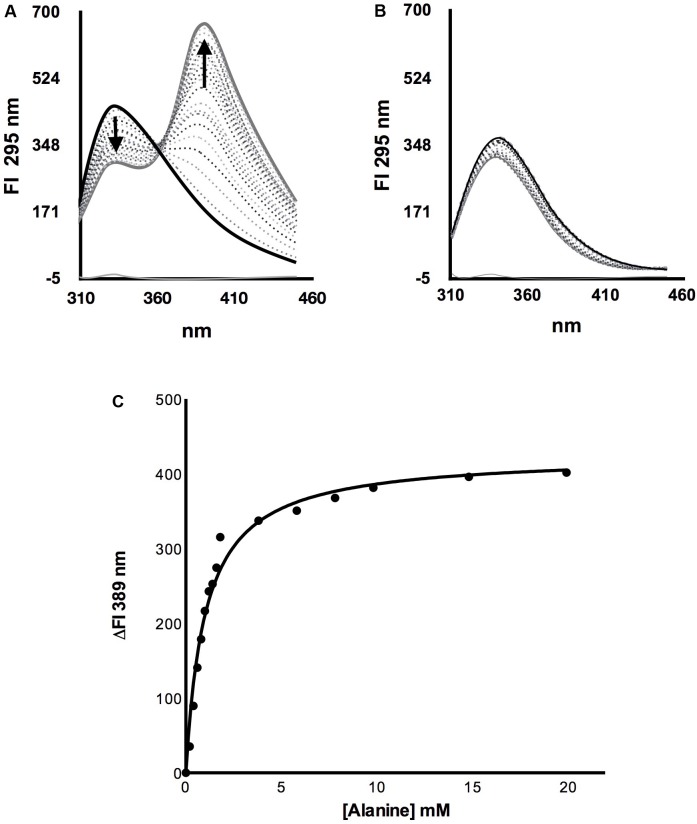
Alanine titration of *Sc*Alt1 and *Sc*Alt2. **(A)** *Sc*Alt1 spectra without alanine is shown in black continuous line. The dotted spectra represent titration with different alanine concentrations, while the gray continuous line follows the spectra obtained with the highest alanine concentration (50 mM). **(B)** In black continuous line *Sc*Alt2 without alanine, dotted spectra represent each one of the various alanine concentrations used for the titration, the gray continuous line follows the spectra obtained with the highest alanine concentration (50 mM). **(C)** Alanine *Sc*Alt1 dissociation constant K_D_ = 1.0 mM ± 0.1 R^2^ 0.98.

To analyze whether *Sc*Alt2 could bind alanine, a titration was performed by adding increasing concentrations of alanine and following the change of intrinsic fluorescence. Both, *Sc*Alt1 and *Sc*Alt2 have three tryptophan molecules, which according to the structural prediction are in equivalent positions. *Sc*Alt1 was used as positive control, since we have clearly established that this enzyme has alanine transaminase activity, indicating that *Sc*Atl1 binds alanine. Two important findings were observed: (a) the fluorescence obtained at 333 nm decreased, and (b) a progressive increase in fluorescence was detected at 389 nm (**Figure 4A**). Fluorescence quenching at 333 nm can be attributed to a conformational change that increases the interactions of tryptophan residues with polar amino acids (Rawel et al., 2006). The increased fluorescence at 389 nm corresponds to energy transfer of the pyridoxal chromophore with some of the chromophores of tryptophan residues (Martínez del Pozo et al., 1989). Interestingly, these changes in fluorescence were not observed when alanine was added to the *Sc*Alt2 preparation (**Figure 4B**), indicating that this protein does not interact with alanine. To determine the dissociation constant for alanine binding, the intrinsic fluorescence changes (ΔFI) of *ScAlt1* at 389 nm were plotted against alanine concentration (**Figure 4C**). The data were fitted to the “One site specific binding” equation shown in Section “Determination of *Sc*Alt1 and *Sc*Alt2 Alanine Binding Affinity,” obtaining a dissociation constant of 1.0 ± 0.1 mM. In addition to alanine, we determined whether *Sc*Alt1 and *Sc*Alt2 could use the rest of the amino acids found in proteins as substrates. We found that in addition to alanine, *Sc*Alt1 could form external Schiff base with glutamate, serine, cysteine, histidine, and aspartic acid; however, for the rest of the amino acids no external Schiff base formation was detected. *Sc*Alt2 was unable to form external Schiff base with either one of the 20 amino acids. Representative results are shown in Supplementary Figure S3.

### *Sc*Alt2 Forms a Schiff Base With PLP

**FIGURE 5:**
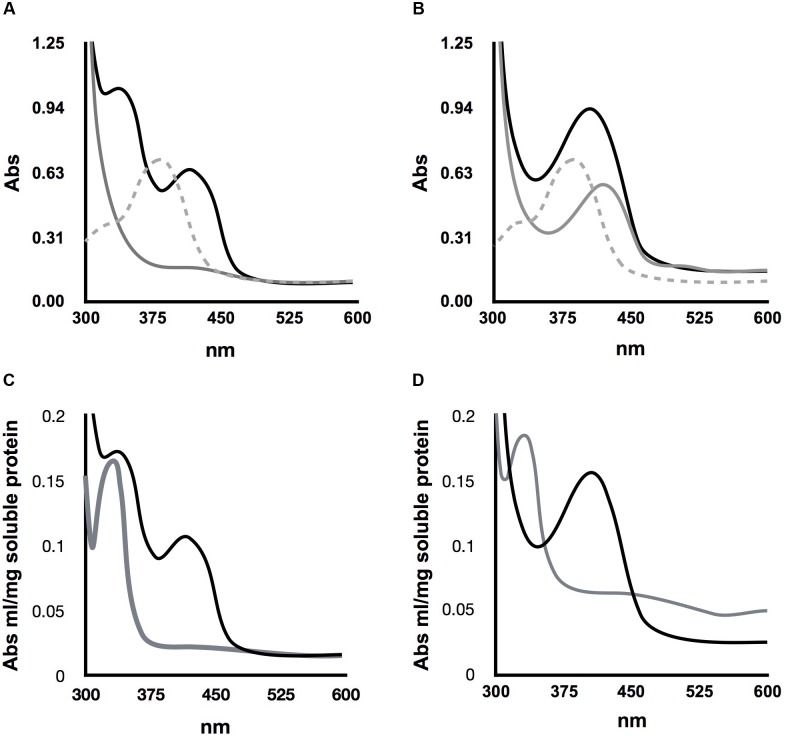
Determination of Schiff base formation in *Sc*Alt1 and *Sc*Alt2. **(A)** Gray line represents *Sc*Alt1 spectra without added PLP, dashed gray line free PLP spectrum and black line represents *Sc*Alt1 spectrum with PLP. Two maximum peaks are observed 330–335 nm and 420 nm in ScAlt1 with PLP. **(B)** Gray line represents *Sc*Alt2 spectra without added PLP, dashed gray line represents free PLP spectrum and black line represents *Sc*Alt2 spectrum with PLP. With or without PLP, a single maximum peak was observed at 420 nm **(C,D)**, reduction of *Sc*Alt1 and *Sc*Alt2 Schiff base after NaBH_4_ addition, resulting in the formation of a single peak at 330 nm. Black line native protein, gray line reduced protein.

A peculiar characteristic of *Sc*Alt2 is its brilliant yellow color, which contrasts with the absence of color of *Sc*Alt1. Considering this observation, we posed the question of whether this difference could be due to different PLP interactions. To address this question, we determined the absorption spectrum from 300 to 600 nm, since in this region the Schiff base can be observed in its different states. Experiments were carried out under two conditions without adding PLP and in the presence of this cofactor. For *Sc*Alt1, no formation of the Schiff base was observed without PLP, however, upon addition of PLP, two maximum absorption peaks were observed being the most prominent the one at 330–335 nm, which should correspond to the enolimine form of the Schiff base, and the less pronounced peak at 420 nm to the ketoenamine derivative of the Schiff base (**Figure 5A**).

For *Sc*Alt2 a maximum peak is observed at 420 nm without PLP, which could correspond to the ketoenimine form of the Schiff base. Upon PLP addition, the 420 nm peak was increased (**Figure 5B**), as compared to that found without PLP (**Figure 5B**). To corroborate the presence of the Schiff base in *Sc*Alt1 and *Sc*Alt2, proteins were incubated with sodium borohydride (NaBH_4_), and in both cases the reported spectroscopic modification for the reduction of the Schiff base was observed, resulting in the formation of a single peak at 330 nm (**Figures 5C,D**).

### *Sc*Alt2 Binding to PLP Is Reversible and Depends on *Sc*Alt2 Structure

**FIGURE 6:**
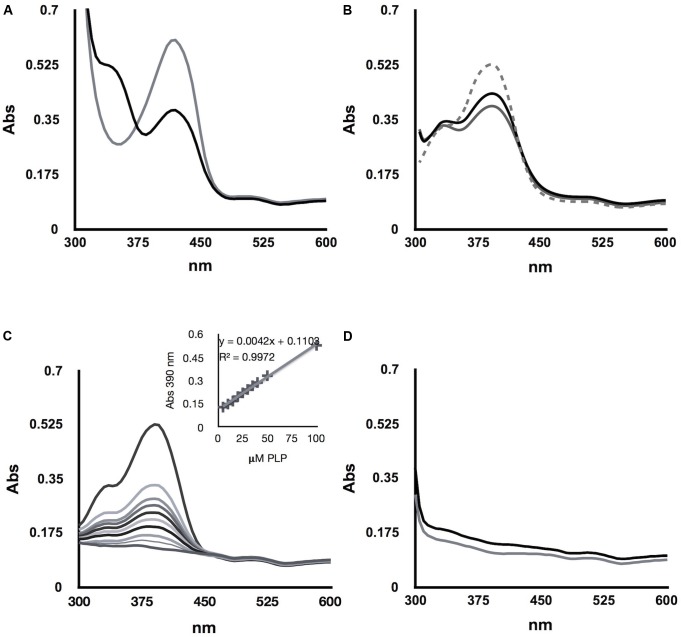
PLP binding is reversible and folding dependent. **(A)** Absorbance of *Sc*Alt1 (black line) and *Sc*Alt2 (gray line) native proteins in UV-visible spectrum. **(B)** Denatured protein spectrum *Sc*Alt1 (black line) and *Sc*Alt2 (gray line) free PLP (gray dashed line). **(C)** PLP calibration curve. **(D)** *Sc*Alt1 and *Sc*Alt2 denatured proteins spectrum without free PLP *Sc*Alt1 (black line) *Sc*Alt2 (gray line).

Pyridoxal phosphate-dependent enzymes represent about 4% of the enzymes reported by the Enzyme Commission, indicating the versatility of action of this cofactor and the important biological role played by PLP (Percudani and Peracchi, 2003). However, all the chemical reactions in which it participates begin with a transimination in which the amino acid substrate displaces the active site lysine residue from the cofactor of the internal Schiff base, to create a new imino linkage between PLP and the substrate, known as external Schiff base (Toney, 2005); thus, a functional Schiff base must allow the transimination reactions. We thus considered the possibility that lack of *Sc*Alt2 alanine transaminase activity could be attributed to the irreversible binding between PLP and *Sc*Alt2. To address this possibility, *Sc*Alt1 and *Sc*Alt2 were first saturated with PLP, then excess PLP was washed, and proteins were then denatured by incubating for 2 h with 6 M Gdn/HCl, at room temperature. A UV-visible absorption spectrum was registered for this samples, and as shown in **Figure 6B**, the maximum absorption peaks of the Schiff base were not observed, as compared to those obtained with the native proteins (**Figure 6A**). When the proteins were separated from the free PLP, it was found that most of the cofactor was liberated from both *Sc*Alt1 and *Sc*Alt2, i.e., 94 and 85%, respectively (**Figure 6C**). Spectrum of denatured *Sc*Alt1 and *Sc*Alt2, without PLP and after dialysis, showed no Schiff base signal (**Figure 6D**). The fact that after denaturation of the proteins PLP was not found bound to either *Sc*Alt1 or *Sc*Alt2 indicates that formation of the Schiff base is reversible and dependent on protein folding.

### *Sc*Alt1 and *Sc*Alt2 Clades Are Conserved in “Sensu Stricto” *Saccharomyces* Strains Showing a Unique and Differential Topology With KLE and ZT Clades

**FIGURE 7:**
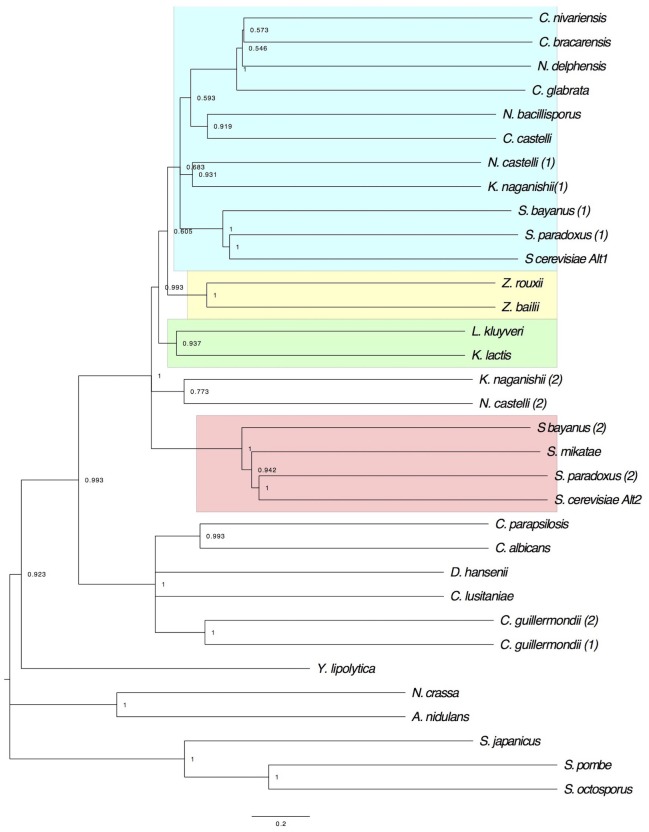
Evolutionary history of Alt proteins: Maximum Likelihood phylogeny of the Alt protein family in yeast. The bootstrap consensus tree inferred from 1000 replicates is taken to represent the evolutionary history of Alts. Branches corresponding to partitions reproduced in less than 50% bootstrap replicates are collapsed. The percentage of replicate trees in which the associated taxa clustered together in the bootstrap test (1000 replicates), are shown next to the branches. *Sc*Alt1 and *Sc*Alt2 associate in independents clades. The different clusters are depicted in boxes: Alt1 (blue box), Alt2 (red box), KLE (green box), and ZT (yellow box).

To analyze the evolutionary history of *Sc*Alt1 and *Sc*Alt2 proteins of *S. cerevisiae*, a phylogenetic tree was constructed with amino acid sequences from representative hemiascomycetes (**Figure 7**). Analysis of the tree revealed three major aspects which should be highlighted: (1) *Sc*Alt1 and *Sc*Alt2 of *S. cerevisiae* were grouped in separate clades with Alt1 and Alt2 of post WGD yeasts. (2) Alts phylogeny supports the hybridization scenarios proposed by Marcet-Houben and Gabaldón (2015), since the Alt1 clade is closer to the parental ZT than to the KLE (**Figure 7**), and it was found grouped with *Saccharomyces sensu stricto* and other yeasts which are not “*sensu stricto*” but which underwent WGD, such as *Candida nivariensis, Candida bracarensis, Nakaseomyces delphensis.* Alt2 forms a distinct clade with “*sensu stricto*” (**Figure 7**), close to the two parental linages ZT and KLE, forming an outgroup, and (3) Alt2 is retained in *sensu stricto*, underscoring the relevance of *Sc*Alt2 retention.

## Discussion

*ScALT2* and *ScALT1* genes arose from the combined action of an interspecies hybridization followed by a WGD event (Kellis et al., 2004; Marcet-Houben and Gabaldón, 2015). Although these paralogs have diversified in various ways, both of them have been retained in the yeast *S. cerevisiae* genome for over 100 million years, although it has been observed that the half-life of a duplicated eukaryotic gene is of approximately 4 million years (Lynch and Conery, 2000). *ScALT1* encodes an alanine transaminase, which conserved the ancestral capacity to biosynthesize alanine and has specialized its catabolic role, constituting an indispensable enzyme for alanine utilization as sole nitrogen source (Escalera-Fanjul et al., 2017). Conversely, *ScAlt2* protein has completely lost its role in alanine metabolism (Peñalosa-Ruiz et al., 2012). Further analysis of *ScALT1* and *ScALT2* specialization has shown that transcriptional regulation of these genes has diversified, resulting in opposed expression profiles: in the presence of alanine, *ALT1* expression is induced while that of *ALT2* is repressed (Peñalosa-Ruiz et al., 2012). Additionally, *Sc*Alt1 and *Sc*Alt2 sub-cellular localization has also diverged, *Sc*Alt1 is in the mitochondria, while *Sc*Alt2 is cytosolic (Peñalosa-Ruiz et al., 2012). Thus, although *Sc*Alt2 has lost its role in alanine metabolism, its peculiar specialization profile, and the fact that it has been retained in several *Saccharomyces* “*sensu strictu*” genomes suggests it carries out a function that could have been selected due to the acquisition of an evolutionary advantage for these yeasts. To determine the structural modifications that led to *Sc*Alt2 loss of function as alanine transaminase, we purified and characterized the *Sc*Alt1 and *Sc*Alt2 proteins. In the case of the *Sc*Alt1, its previously described role as alanine transaminase (Peñalosa-Ruiz et al., 2012) was confirmed, and its kinetic characterization showed that it has the characteristic ping-pong mechanism displayed by transaminases. It was additionally found that the kinetic parameters of *Sc*Alt1 are similar to those reported for the orthologous proteins *Kl*Alt1 and *Lk*Alt1 (Escalera-Fanjul et al., 2017).

On the other hand, *ScAlt2* structural analysis raised an interesting paradox: it turns out that this protein has retained the catalytic residues that characterize alanine transaminases; however, it does not exhibit this catalytic capacity. In order to find structural differences between *Sc*Alt1 and *Sc*Alt2 proteins, models of each one of these proteins were developed and analyzed. Our results show that both proteins display similar structures, indicating that for these proteins, molecular modeling might not allow finding structural modifications that could explain their different enzymatic capacities, which could be due to thermodynamic and kinetic folding variability (Pey et al., 2013).

Since *Sc*Alt2 lack of activity as alanine transaminase could not be explained using molecular modeling, it was decided to perform *Sc*Alt1 and *Sc*Alt2 structural characterization. When analyzing *Sc*Alt1 and *Sc*Alt2 secondary structure by CD, it was found that both proteins had similar secondary structure, ruling out the possibility that *Sc*Alt2 was a destructured protein. In spite of this, fluorescence and quenching experiments showed that when *Sc*Alt2 was compared with *Sc*Alt1, it showed a higher exposure of the hydrophobic residues to the solvent, indicating that *Sc*Alt2 has a more expanded structure as compared to that of *Sc*Alt1, in agreement with the fact that *Sc*Alt2 is more thermolabile than *Sc*Alt1. When analyzing the quaternary structure by molecular exclusion chromatography, we found that like most transaminases, both *Sc*Alt1 and *Sc*Alt2 are dimeric enzymes (Eliot and Kirsch, 2004).

To further analyze *Sc*Alt2 capacity to participate in alanine metabolism, and considering that *Sc*Alt1 and *Sc*Alt2 belong to the PLP dependent fold type I proteins, which undergo conformational changes from an open state (enzyme without ligand) to a closed state (enzyme with ligand) (Raboni et al., 2010), we asked whether *Sc*Alt2 was able to bind alanine, following the fluorescence spectra as a measure of conformational changes. When *Sc*Alt1 was titrated with alanine, we found a decrease in fluorescence at 333 nm and the appearance of a maximum at 389 nm. The decrease in fluorescence at 333 nm could be attributed to the modification of the tertiary structure caused by tryptophan interaction with charged amino acids, while the appearance of the maximum at 389 nm should correspond to the interaction of the indole group of a tryptophan with the PLP aromatic ring (Martínez del Pozo et al., 1989). However, in the case of *Sc*Alt2 no change in the intrinsic fluorescence spectrum was observed. It can be thus concluded that there is no conformational change when *Sc*Alt2 is titrated with alanine, indicating that this protein does not bind this amino acid. It is possible that having an expanded structure *Sc*Alt2 distorted its binding site, resulting in the loss of alanine transaminase activity.

It is known that PLP-dependent proteins require that the cofactor binds in the form of a Schiff base (Toney, 2005). Therefore, another possibility that could explain the lack of *Sc*Alt2 transaminase activity could be due to the fact that it is not capable of binding PLP in the form of Schiff base. To analyze this possibility, changes in the absorption spectrum between 300 and 600 nm, were followed, since in this region the different forms of the Schiff base can be observed. Additionally, reduction of the Schiff base with sodium borohydride (NaBH_4_) was followed. It was found that both, *Sc*Alt1 and *Sc*Alt2 have PLP attached through a Schiff base, since they show the corresponding absorption maxima, and when adding NaBH_4_ the characteristic spectrophotometric modification was observed (**Figures 5A–D**) (Hughes et al., 1962).

**FIGURE 8:**
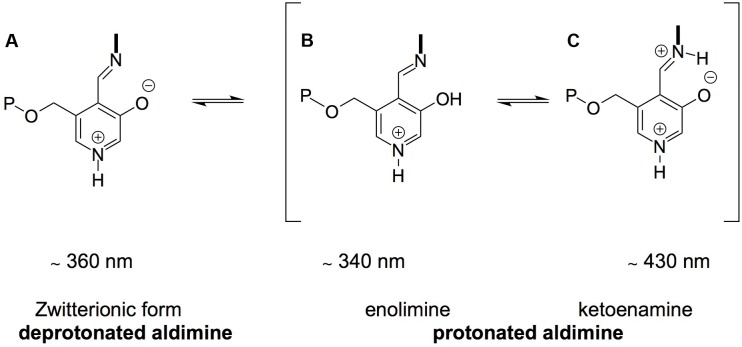
Structure of PLP Schiff Base. **(A)** Deprotonated aldimine, **(B,C)** represent the protonated aldimine tautomers: enolimine and ketoenamine.

Although *Sc*Alt1 and *Sc*Alt2 bind PLP as a Schiff base, a difference was observed in the tautomeric forms. *Sc*Alt1 presents two maxima in the UV-visible spectra, which represent the protonated Schiff base in its two tautomeric forms, ketoenamine and enolimin, having a more abundant enolimin form (**Figure 8**). In the case of *Sc*Alt2, the Schiff base only appears in the ketoenimin form. It is known that the formation of the enolimin tautomer is favored in non-polar environments, while in polar environments the formation of ketoenimin tautomer is preferred (Hidalgo et al., 1994).

A difference in the surrounding environment of PLP could explain why *Sc*Alt2 does not have alanine transaminase activity, according to the stereoelectronic theory posed by Dunathan (1971). A specific geometry is necessary for enzyme catalysis that places or orientates the bond to be broken perpendicular to the plane of the π system (Dunathan, 1971). Therefore, a change in PLP immediate environment causes a modification in the specific geometry that inhibits the reaction. An example of this phenomenon is the case of the β_2_ subunit of tryptophan synthase, which has the ketoenamin form as an active tautomer. When the temperature is raised, the formation of the enolimin tautomer is favored and the catalytic activity is lost (Ahmed et al., 1996).

The theory of electronic modulation through protonation considers that PLP-dependent enzymes are able to optimize specific reactions by modulating PLP electronic states, through changes in the active site environment. It has been shown that by disturbing the hydrogen bond network that interacts with PLP the catalysis of the enzyme is affected (Dajnowicz et al., 2017). In the case of *Sc*Alt2, it was shown that the solvent has higher access to tryptophan, which led us to propose that the solvent has greater access to the active site. It is known that hydrogen bonds between residues and solvent are favored instead of the formation of hydrogen bonds between residues (Fleming and Rose, 2005); therefore, it is possible that the hydrogen bond network surrounding the *Sc*Alt2 catalytic site is modified resulting in lack of alanine transaminase activity.

To determine whether PLP binding to *Sc*Alt2 was reversible and dependent on folding, *Sc*Alt1 and *Sc*Alt2 purified samples which carry the tightly bound PLP, were denatured with Gdn/HCl and free PLP was quantified. For both proteins, we found that most of the PLP was released by denaturing the protein, indicating that PLP binding is fold-dependent, and that as previously shown, the preservation of the cofactor binding domain is required (Denessiouk et al., 1999).

The obligated common step in reactions catalyzed by PLP-dependent enzymes is transimination, in which the lysine that binds PLP is released, and PLP forms a new imino bond with the substrate, generating the external Schiff base (Toney, 2005). Therefore, a catalytically competent Schiff base must display reversibility. As previously mentioned, when *Sc*Alt1 and *Sc*Alt2 were denatured, both proteins released the PLP. We can thus conclude that cofactor binding in both enzymes is reversible, allowing the possibility that *Sc*Alt2 could have a PLP-dependent catalytic activity.

Finally, the evolutionary history of *Sc*Alt1 and *Sc*Alt2 proteins was analyzed, finding that they are grouped into different clades and their affiliation with ZT and KLE is also different, as was previously observed (Escalera-Fanjul et al., 2017). In the case of *Sc*Alt1, it has a close relationship with ZT, while *Sc*Alt2 is positioned in the tree as an outgroup of ZT and KLE, constituting a clade with “*sensu strictu*” yeasts. *Saccharomyces* “*sensu stricto*” is a species complex that includes most of the yeast strains displaying fermentative and respiratory lifestyles. The difference in the relationship between ZT and KLE, according to Marcet-Houben and Gabaldón (2015), is a reflection of their similarity with the parental clades. It can be considered that the *Sc*Alt1 clade, has retained a greater similarity with ZT, while the *Sc*Alt2 clade retained characteristics of both ZT and KLE constituting a hybrid protein. It will be necessary to study the orthologous genes found in species related to ZT and KLE in order to learn more about *Sc*Alt2 physiological role.

Although dependent PLP enzymes can only be organized in around six different folding types and have been studied for decades; determining their function by exclusively using the homology criterion is complicated since the mechanical characteristics of the PLP catalysis can facilitate either convergent or divergent evolution. In the first case, the appearance of enzymes with identical or very similar activities along independent lineages could be observed. In the second case, divergent evolution, from the same common ancestor can lead to the acquisition of very different activities (Christen and Mehta, 2001). It is therefore very likely that *Sc*Alt2 has diverged through the modification of its interaction with PLP, which affected its specificity and even the type of reaction it can perform. To determine its function, it will be necessary to explore different substrates and analyze its crystallographic structure.

## Conclusion

*Sc*Alt1 and *Sc*Alt2 proteins have a similar structure; however, *Sc*Alt2 has a more expanded conformation as compared to that of *Sc*Alt1, resulting in a different mode of interaction with PLP, which could result in lack of alanine transaminase activity. The fact that *Sc*Alt2 forms a catalytically competent Schiff base, and the selective retention of the Alt2 clade along the “*sensu stricto*” yeasts for more than 100 million years in addition to the evolutionary patterns observed for PLP-dependent enzymes, make us propose the possibility that Alt2 proteins have a yet undescribed function.

## Author Contributions

AG designed the experiments, wrote the MS, obtained yeasts, contributed reagents and materials. ER-O conceived, designed, performed the experiments, and wrote the MS. BA-L performed and designed the experiments. HR-V designed the experiments and contributed to MS. MG-A performed and designed the experiments. JC-B and JP designed the experiments.

## Conflict of Interest Statement

The authors declare that the research was conducted in the absence of any commercial or financial relationships that could be construed as a potential conflict of interest. The reviewer EL-R and handling Editor declared their shared affiliation.

## Footnotes

1 https://portals.broadinstitute.org/regev/orthogroups/

2 http://phylomedb.org/?q=about

3 http://www.megasoftware.net/

4 https://blast.ncbi.nlm.nih.gov/Blast.cgi
