# Supplementary material for: Saccharomyces cerevisiae Differential Functionalization of Presumed ScALT1 and ScALT2 Alanine Transaminases Has Been Driven by Diversification of Pyridoxal Phosphate Interactions
Source: Front Microbiol. 2018 May 14;9:944. doi: 10.3389/fmicb.2018.00944 (PMC5960717; doi:10.3389/fmicb.2018.00944)
Supplement: Supplementary file 1 [file Image_1.PDF]

Saccharomyces cerevisiae differential functionalization of presumed *ScA*lt1 and *ScA*lt2 alanine transaminases has been driven by pyridoxal phosphate interaction diversification

Authors:
Erendira Rojas-Ortega, Beatriz Aguirre, Horacio Reyes-Vivas, Martín González-Andrade, Jose Carlos Campero-Basaldúa, Juan Pablo Pardo and Alicia González*

*Author for correspondence:

Alicia González

amanjarr@ifc.unam.mx

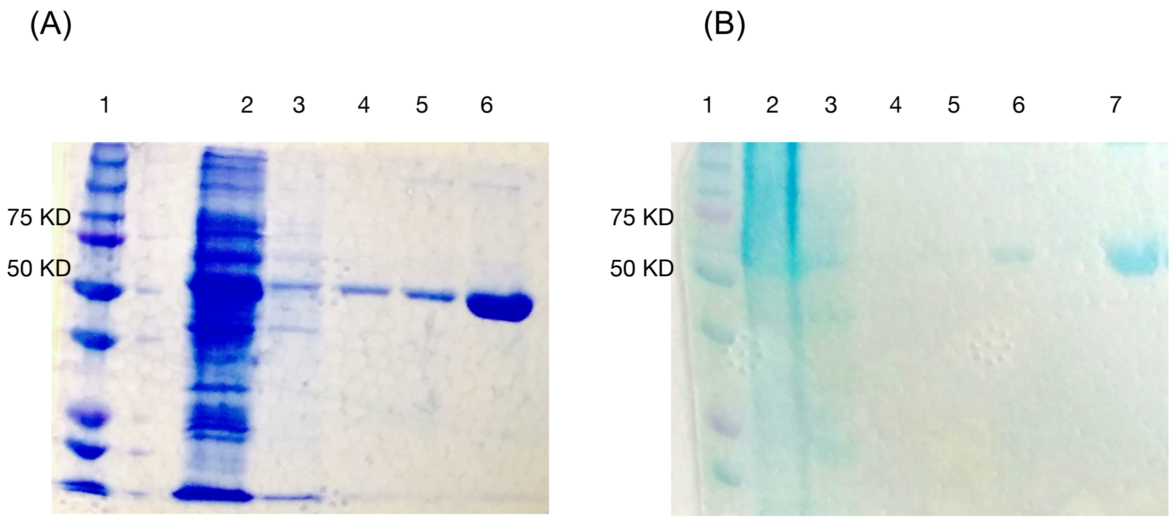

Figure S1. *Sc*At1 and *Sc*Alt2 purification. Recombinant enzymes from *S. cerevisiae* were produced in *E. coli*. A) *Sc*Alt1 (61 kDa): Lane 1, Ladder; Lane 2, Soluble fraction, Lane 3 washing 30 mM; Lane 4, washing 50 mM; Lane 5, washing 80 mM; Lane 6, elution 300 mM imidazol. B) *Sc*Alt2 (59 kD): Lane 1, Ladder; Lane 2, Soluble fraction Lane 3, washing lysis buffer; Lane 4; washing 30 mM; Lane 5, washing 50 mM; Lane 6, washing 80 mM; Lane 7, elution 300 mM imidazol.
