# Supplementary material for: Saccharomyces cerevisiae Differential Functionalization of Presumed ScALT1 and ScALT2 Alanine Transaminases Has Been Driven by Diversification of Pyridoxal Phosphate Interactions
Source: Front Microbiol. 2018 May 14;9:944. doi: 10.3389/fmicb.2018.00944 (PMC5960717; doi:10.3389/fmicb.2018.00944)
Supplement: Supplementary file 2 [file Image_2.PDF]

# *Saccharomyces cerevisiae* differential functionalization of presumed *ScA*lt1 and *ScA*lt2 alanine transaminases has been driven by pyridoxal phosphate interaction diversification

Authors:
Erendira Rojas-Ortega, Beatriz Aguirre, Horacio Reyes-Vivas, Martín González-Andrade, Jose Carlos Campero-Basaldúa, Juan Pablo Pardo and Alicia González*

*Author for correspondence:

Alicia González

amanjarr@ifc.unam.mx

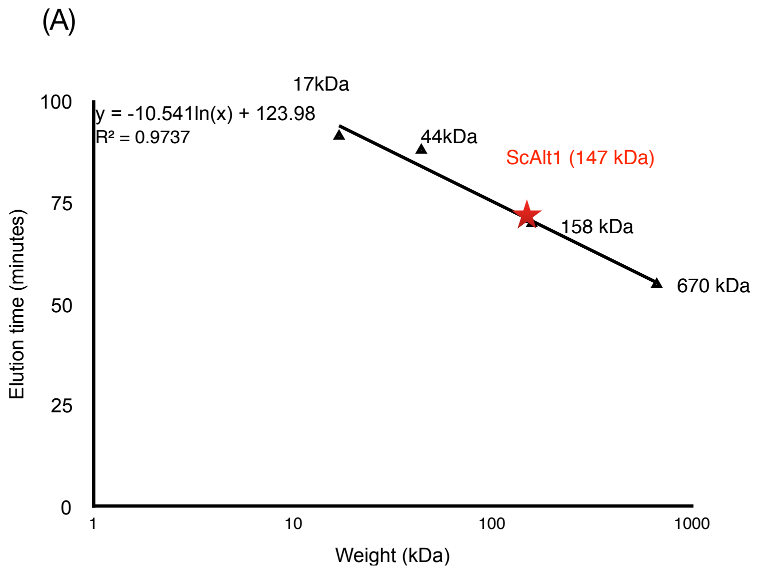


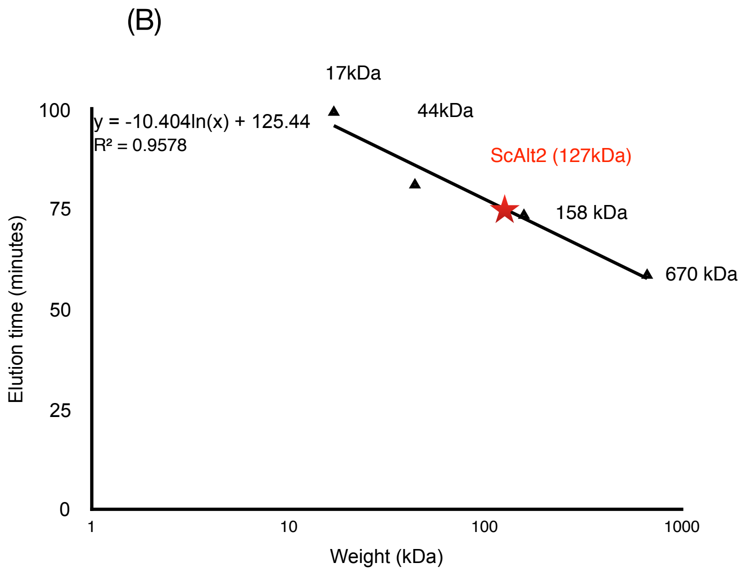


| (A) | Protein | Weight KDa | Elution time (minutes) |
|---|---|---|---|
| Standard | Thyroglobulin (bovine) | 670 | 55 |
| Standard | γ-globulin (bovine) | 158 | 70 |
| Standard | Ovalbumin (chicken) | 44 | 88 |
| Standard | Myoglobin (horse) | 17 | 92 |
| | **ScAlt1** | 147 | **71** |

| (B) | Protein | Weight (KDa) | Elution time (minute) |
|---|---|---|---|
| Standard | Thyroglobulin (bovine) | 670 | 59 |
| Standard | γ-globulin (bovine) | 158 | 74 |
| Standard | Ovalbumin (chicken) | 44 | 81 |
| Standard | Myoglobin (horse) | 17 | 99 |
| | **ScAlt2** | 127 | **75** |

**Figure S2. ScAlt1 and ScAlt2 are organized as dimers as determined by size exclusion chromatography.** A) ScAlt1 and B) ScAlt2 oligomeric state calibration curves: black triangles represent proteins used as standards, red stars alternatively show ScAlt1 or ScAlt2. Molecular weight and elution time of proteins used as standards are shown in the tables A and B.
