# Supplementary material for: Saccharomyces cerevisiae Differential Functionalization of Presumed ScALT1 and ScALT2 Alanine Transaminases Has Been Driven by Diversification of Pyridoxal Phosphate Interactions
Source: Front Microbiol. 2018 May 14;9:944. doi: 10.3389/fmicb.2018.00944 (PMC5960717; doi:10.3389/fmicb.2018.00944)
Supplement: Supplementary file 3 [file Image_3.PDF]

Saccharomyces cerevisiae differential functionalization of presumed *ScA*lt1 and *ScA*lt2 alanine transaminases has been driven by pyridoxal phosphate interaction diversification

Authors:
Erendira Rojas-Ortega, Beatriz Aguirre, Horacio Reyes-Vivas, Martín González-Andrade, Jose Carlos Campero-Basaldúa, Juan Pablo Pardo and Alicia González*

*Author for correspondence:

Alicia González

amanjarr@ifc.unam.mx

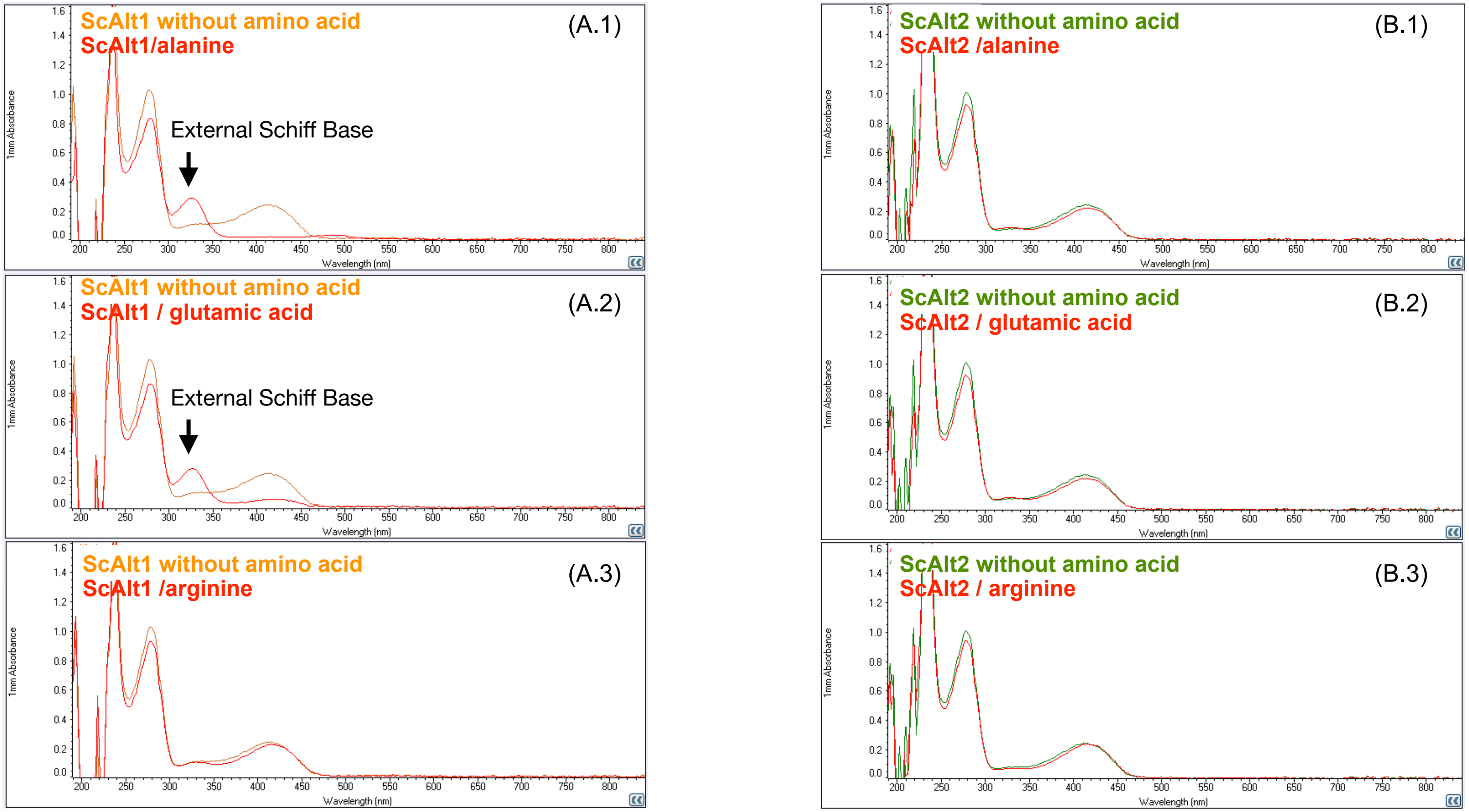

Figure S3. ScAlt1 and ScAlt2 show diverse amino acid interaction patterns. Representative spectra of ScAlt1 (A.1-A.3) and ScAlt2 (B.1-B.3) after addition of different amino acids. ScAlt1 and ScAlt2 exhibit three absorbance maximums, 228 nm (free PLP), 280 nm (ScAlt1 or ScAlt2), 390 nm (free PLP and internal Schiff base). The red and orange lines respectively represent ScAlt1 with or without alanine (A.1) or glutamic acid (A.2), at PLP saturation conditions. Formation of external Schiff base results in the spectrum shift observed in the presence of alanine or glutamic acid at a maximum at 325 nm (A.3). The red and orange lines respectively represent ScAlt1 with or without arginine, at PLP saturation conditions. No spectrum shift is observed in the presence of arginine, meaning that ScAlt1 does interact with arginine. B.1-3) The red and green lines respectively represent ScAlt2 with and without alanine (B.1), glutamic acid (B.2) or arginine (B.3) at PLP saturation conditions. No spectrum shift is observed indicating that ScAlt2 does not interact with these amino acids.
